# The effect of a split portion of flaxseed on 24-h blood glucose response

**DOI:** 10.1007/s00394-020-02333-x

**Published:** 2020-07-22

**Authors:** Awatif Almehmadi, Helen Lightowler, Magali Chohan, Miriam E. Clegg

**Affiliations:** 1 Department of Sport, Health Sciences and Social Work, Faculty of Health and Life Sciences, Oxford Brookes University, Oxford, OX3 0BP UK; 2 Department of Clinical Nutrition, College of Applied Medical Sciences, Umm Al-Qura University, 21421, Makkah, Saudi Arabia; 3 Department of Health and Exercise Science, Faculty of Sport, Health and Applied Science, St Mary’s University, Twickenham, TW1 4SX UK; 4 Department of Food and Nutritional Sciences, School of Chemistry, Food and Pharmacy, University of Reading, Reading, RG6 6AP UK

**Keywords:** Flaxseed, Linseed, Blood glucose, Continuous glucose monitor, Blood glucose incremental area under the curve (iAUC), 24 blood glucose profile

## Abstract

**Purpose:**

Flaxseed can be effective at lowering and stabilising blood glucose responses. The aim of this study was to determine whether flaxseed could lower blood glucose response more effectively when consumed as a single portion of 30 g, or a split portion consumed three times per day (10 g flaxseed per portion).

**Methods:**

The study was a randomised, repeated measures, cross-over design. Fifteen healthy participants consumed either (1) three flaxseed muffins containing a total of 30 g of flaxseed once in the morning, (2) three flaxseed muffins consumed at three different timepoints across the day (10 g flaxseed per muffin) or (3) three control muffins consumed at three different timepoints across the day (0 g flaxseed). The 24-h blood glucose response was measured using a continuous glucose monitor.

**Results:**

The results of this study demonstrated that flaxseed muffins given three times a day were effective at lowering and maintaining blood glucose levels over 24 h, compared to the control muffins and that both flaxseed treatments resulting in a lower blood glucose iAUC during the night.

**Conclusion:**

The results of this study indicated that adding flaxseed to a daily diet produced a lower glucose profile over 24 h in a free-living context compared to the control muffins.

## Introduction

There is a considerable body of evidence indicating that the consumption of low glycemic index (GI) foods reduces blood glucose fluctuations, and benefits the management and prevention of diabetes and prediabetes [1–3]. Low GI foods help to stabilise blood responses at a normal level for longer period of time [4] minimising potentially large fluctuations in blood glucose levels [2, 5]. Epidemiological evidence suggests that postprandial blood glucose levels are more strongly related to cardiovascular events than fasting blood glucose in individuals with diabetes [6, 7]. Current eating patterns can mean that a high proportion of the day is spent in the postprandial state, which produces the highest plasma glucose levels [8].

Foods which contain a high amount of fibre generally have a lower postprandial blood glucose and insulin response, a lower GI, and are more effective at preventing type two diabetes in healthy individuals [1]. Flaxseed is considered to be one of the key plant sources of dietary fibre, as it contains both soluble and insoluble fibre with a ratio of soluble to insoluble varying between 20:80 and 40:60. The soluble fibre in flaxseed is mucilage gum and insoluble fibre is cellulose, hemicellulose and lignin [9, 10].

Previous studies have demonstrated that flaxseed can be effective at lowering and stabilising the blood glucose response in healthy, pre-diabetic and diabetic individuals [11–15]. The question remains as to whether flaxseed is more effective at maintaining blood glucose at the normal level over 24 h when the portion is given once or spread out throughout the day. The majority of studies on flaxseed and glycaemic response gathered the data in a laboratory setting the morning after an overnight fast, which does not represent the physiological responses across a full day.

Continuous glucose monitoring systems (CGMS) provide a method of evaluating subcutaneous interstitial fluid glucose over a number of days, by applying an electrochemical detector under free-living conditions [16]. CGMS evaluates interstitial glucose concentrations every 5 min and are minimally invasive, which gives an opportunity to evaluate the direction, magnitude, duration, frequency and causes of fluctuations in blood glucose levels [16, 17]. It can give a detailed view of the blood glucose profile, which can potentially show when and to what extent, an intervention affects the glycaemic response, while allowing the participants to continue their daily lives and activities uninterrupted [5, 18].

Currently, to best of our knowledge, no study has investigated the effect of splitting a portion of flaxseed on blood glucose control, or whether the flaxseed is more effective administered in a single or split portion. In the current study, the flaxseed portion was split over the three meals, which may cause a lower blood glucose post each of the three meals. The aim of this study, therefore, was to determine the most effective timing of flaxseed consumption to lower blood glucose response in healthy participants. This study used a single and a split portion regimen and investigated the blood glucose response over a 24-h period using a CGMS to determine whether flaxseed was more effective when consumed as a single portion of 30 g or as a split portion consumed three times per day (10 g flaxseed per portion).

## Materials and methods

### Participants

Healthy male and female adults aged between 18–60 years were recruited into this study at Oxford Brookes University through advertisements containing the researcher contact details between March 2017 and November 2017. The inclusion criteria were; aged 18–65 years; Body mass index (BMI) ≤ 30 kg/m^2^; fasting blood glucose < 6.1 mmol/l; not-pregnant or lactating; no known diabetes or impaired glucose tolerance; no medical condition(s) or medication(s) known to affect glucose regulation or appetite and/or which influence digestion and absorption of nutrients; no major medical or surgical event requiring hospitalisation within the preceding three months; and no food allergy or intolerance to any of the study foods. Ethical approval for the study has been obtained from the University Research Ethics Committee at Oxford Brookes University (UREC Registration No: 161046).

### Study design

The study was a randomised, cross-over repeated measures design. Participants undertook seven visits in total, the first was the screening visit, three initial sessions for sensor insertion and three follow-up sessions for sensor removal.

### Consent and screening

During the screening visit, participants were asked to sign a consent form and to complete a health questionnaire, which included questions regarding to food allergies/intolerances, metabolic diseases, smoking habits, physical activity, medical conditions and medication. After completing the questionnaire, the following anthropometric measurements were taken: height using a free-standing stadiometer (SECA, Germany), body weight, body fat percent and fat-free mass using the Tanita body composition analyser (BC-418, Tanita UK Ltd, Middlesex, UK). Height was recorded to the nearest 0.1 cm and body weight was recorded to the nearest 0.1 kg. If the participants were eligible after taking the anthropometric measurements and did not have any of the exclusion criteria, then a fasting (overnight for 12 h) blood glucose measurement was taken from a finger-prick blood sample to determine the baseline data for glucose and if the participant was eligible to participate in the study.

### Test days

At the initial sessions the CGMS sensor was inserted under the skin in the abdominal region between 15.00–17.00 h the day before the test day. Participants collected food for the test day, as well as prepared food for an evening meal the day before the test day. This ensured all participants began the test day following a similar meal. There was no need to fast for the sensor insertion session and there were no restrictions on the types of clothes that could be worn.

Participants were required to remain at the Oxford Brookes Centre for Nutrition and Health for 1 h to allow the sensor to initialise after trained personnel inserted the sensor. The participants were required to take four capillary finger-prick blood glucose readings using a single use lancing system (Unistik 3, Owen Munford, Woodstock, UK). The readings needed to be taken one and 2 h after the insertion of the sensor, before dinner and before bedtime. The purpose of the capillary finger-pricks was to calibrate the CGMS sensor.

All participants were provided with a finger-prick blood glucose monitoring device (ACCU-CHEK^®^ Performa Nano, Roche Diabetes Care Limited, Burgess Hill UK). Participants were provided with written instructions on using the device and performing a capillary finger-prick; all participants were also trained to carry on taking blood glucose readings by themselves while the sensor was inserted, participants were required to avoid alcohol and restrict intense physical activity (e.g., long periods at the gym, running, aerobics). Dinner on pre-test day needed to be consumed between 18.00 and 20.00 h.

On the test day which came the day after sensor insertion, the participants were required to take four capillary finger-pricks to measure blood glucose readings following the same procedure as the day prior the test day (before each main meal and before bedtime). Participants were aware of how to follow the correct procedures to dispose of the waste safely and they were provided with a clinical waste bin to dispose of all the items after use.

The participants were instructed about when to take their meals on the test day; breakfast between 7.00 and 9.00 h, the first snack between 10.00 and 11.00 h, lunch between 12.00 and 14.00 h, the second snack between 16.00 and 17.00 h and dinner between 18.00 and 20.00 h. They were told not to consume any food or drink which was not provided, apart from water and hot drinks such as tea, coffee or herbal tea. They were asked to record all drinks consumed.

On the three test days: participants randomly consumed either control or flaxseed muffins in one of the following orders: (1) Flaxseed once a day: three ground flaxseed muffins with breakfast, one control muffin with lunch and dinner; (2) Flaxseed 3 times a day: one ground flaxseed muffin and two control muffins at breakfast and one flaxseed muffin at lunch and dinner; (3) Control muffins three times a day: three control muffins at breakfast and one control muffin at lunch and dinner.

On the follow-up sessions the participants attended the Oxford Brookes Centre for Nutrition and Health in the morning between 7.00 and 9.00 h after an overnight fast for the CGMS sensor removal. One final capillary finger-prick glucose reading was taken to calibrate the sensors before removing. There was a washout of at least 1 week between the test days. The wash out period was needed to minimise the carry over effect between the different treatments and was based on previous similar studies [19, 20].

### Continuous glucose monitoring system sensor

Measurement of the 24-h glucose response was made using the Medtronic MiniMed iPro^®^2 Professional Continuous Glucose Monitoring (Northridge, CA, USA) which recorded glucose levels every 5 min, giving a total of 288 readings over 24 h. The CGMS is a technology that has been previously used in other studies [21, 22]. This technology provides a full view of the glycaemic level every 5 min, to potentially show when and to what extent, an intervention affects the glycaemic response. It also allows the participants to continue their daily lives and activities uninterrupted [21, 23, 24]. O’Riordan et al. [25] found that the CGMS performance on two occasions over a 12-month period were reliable, reproducible and repeatable.

Using an applicator or self-insertion device, a thin plastic sensor is inserted just under the skin of the abdomen by trained personnel. The receiver can store information for later use and long-term data can be downloaded to a computer programme CareLink iPro Software, which is a Web-based system, designed to generate reports and store data [26, 27].

### Muffins

The muffins (flaxseed and control) were prepared following a standard recipe and the flaxseed muffins were prepared by replacing 48% of the flour in the control muffins with ground flaxseed each flaxseed muffins contain 10 g ground flaxseed. A 150 g sample of each muffin type was analysed for nutritional content by Eurofins Scientific Food Testing Ltd. (Wolverhampton, UK) (Table 1). To prepare the muffins, first the dry ingredients (flour, sugar, baking soda, baking powder, nutmeg and cinnamon) were sifted and thoroughly mixed together with ground flaxseed if required. The banana was mashed and the liquid ingredients (semi-skimmed milk, butter, vanilla and eggs) were mixed in before adding to the dry ingredients. All muffin mixtures were placed into non-stick petite muffin cups tray and baked at 190 °C (375 ºF) in a pre-heated oven for 20 min. After baking, the muffins were allowed to cool for 10 min before they were then removed from the tray. Then they removed from the tray for complete cooling on a wire rack for 30 min.

**Table 1:** Muffin formulation for twelve muffins and nutrient profile per 100 g

Ingredients	Muffin formulation		
	Unit	Control	Flaxseed
Melted butter	g	75	75
Self-rising flour	g	250	130
Flaxseed	g	–	120
Baking powder	g	5	5
Bicarbonate of soda	g	2.5	2.5
Ground cinnamon	g	2.5	2.5
Nutmeg	g	2.5	2.5
Caster sugar	g	115	115
Vanilla extract	ml	5	5
Large ripe bananas	g	240	240
Medium eggs	g	106	106
Semi skimmed milk	ml	125	125

Ingredients	Nutrient profile/100 g	
	Control	Ground flaxseed
Crude protein (g)	5.5	7.2
Carbohydrate (avail) (g)	40.9	30.8
Total sugars (g)	18.7	18.2
Total fat (g)	8.5	14.9
Total fibre (AOAC) (g)	1.6	4.9
Energy (kcal)	265	296
Energy (kJ)	1120	1240
Saturated fatty acids (g)	4.8	6.9

### Dietary intervention

The participants were provided with pre-packaged meals for the evening meal before the test day and all meals for the test days. The meals were designed to meet the dietary reference values for energy and macronutrients for males and females based on the recommendations of the Scientific Advisory Committee on Nutrition [28]. The meals and snacks were made up from control or flaxseed muffins, orange juice, Greek style yoghurt, apple, hummus sandwich, green salad, vegetable lasagne, low fat berry medley yoghurt, nuts, orange, grapes and an oat and honey bar. Each food was labelled with the time that it should be consumed, as well as the meal type.

All meals were planned using Nutritics software ver 5.022 (Nutritics LTD, Dublin, Ireland) based on for a healthy standard adult males and females; if the meal components were not on the Nutritics database, all the energy and macronutrient information which was needed to plan the meals for each test day was inserted manually according to the nutrition facts label on that product.

### Analysis of continuous glucose monitoring system data

All data from the sensors were downloaded to the CareLink iPro Software (MiniMed, Fridley, Minnesota) for each participant, which was coded with the participant’s number and the test number; all capillary results for blood glucose levels were inserted manually for each test treatment. All the times that the meals were consumed were inserted manually and all the data were then exported to Microsoft Excel (Windows version 2010) to complete the data analysis. One day (24-h data with 288 glucose readings) was determined to be from 06:00 on day 1 to 06:00 on day 2. For each test day, the following measurements were performed: Mean blood glucose concentration for 24 h, day (06.00–22.00 h), night (22.00–06.00 h) and pre–post each main meal was recorded. The blood glucose iAUC was also calculated geometrically for each participant’s blood glucose response with the 06.00 h glucose result used as the baseline. Blood glucose peak values for each main meal, as well as the time to peak were also recorded. The standard deviation calculated for all above measurements, except for mean blood glucose concentration over 24 h, day and night, where standard error was calculated.

### Gastrointestinal symptoms

A gastrointestinal symptoms questionnaire was provided to be filled in after each meal for 24 h after each test day. The questionnaire was used to assess four different gastrointestinal symptoms nausea, abdominal cramping, distension and flatulence. Participants rated the frequency and the severity of those symptoms. The scales were used to assess frequency (100 mm = ‘much more often than normal’ to 0 mm = ‘not more often than normal’) and severity (100 mm = ‘very severe’ to 0 mm = ‘none’) of the symptoms [26].

### Statistical analysis

The required sample size for this study was estimated to be 15 to be able to detect a 5% difference between 3 days in the overall blood glucose iAUC with the confidence level as 95% and for a medium effect size, with α-0.05 (two-tailed) using G*power (Version 3.0.10). The power of study was 90% and the number of participants was based on the previous studies [29, 30].

Statistical analysis was performed using SPSS software, (Statistical Package for the Social Sciences) version 25, (Chicago, USA). Prior to statistical analysis, the normality of the data was assured using the Shapiro–Wilks statistical test to investigate the normality of the data; where data were not normally distributed, non-parametric tests were used.

All results were reported as the mean ± SD or SEM (not normally distributed data). Differences in mean blood glucose concentration, time to peak, iAUC and gastrointestinal symptoms between the three test days were assessed using repeated measures ANOVA followed by Bonferroni correction for multiple comparisons if the data were parametric, or the Friedman test and Wilcoxon tests for non-parametric data. *p* < 0.05 was considered statistically significant.

The Pearson correlation coefficient and the method Bland–Altman technique [31] were used to assess the correlation and agreement between the CGMS sensor data and ACCU-CHEK^®^data.

## Results

### Participants characteristics

Fifteen healthy adults (ten female, five male) were recruited (Table 2).

**Table 2:** Baseline characteristics of fifteen participants (mean ± SD)

	All (*n* = 15)	Male (*n* = 5)	Female (*n* = 10)
Age (y)	30.3 ± 9.1	25.0 ± 3.8	32.9 ± 10.0
Height (m)	1.7 ± 0.1	1.8 ± 0.1	1.6 ± 0.8
Weight (kg)	69.7 ± 13.3	81.4 ± 8.4	63.8 ± 11.8
BMI (kg/m^2^)	24.1 ± 2.9	24.6 ± 2.8	23.8 ± 3.1
Body fat%	25 ± 9.3	18.2 ± 5	29.4 ± 8.9
Fasting blood glucose (mmol/l)	5.0 ± 0.3	5.0 ± 0.5	5.0 ± 0.3

### Comparison of continuous glucose monitoring system and the capillary blood glucose readings

The results showed there was a strong correlation observed between the CGMS and the capillary blood finger-pricks carried out using Accu-Chek for the 405 paired glucose readings (*r* = 0.57, *p* < 0.001).

**Fig. 1:**
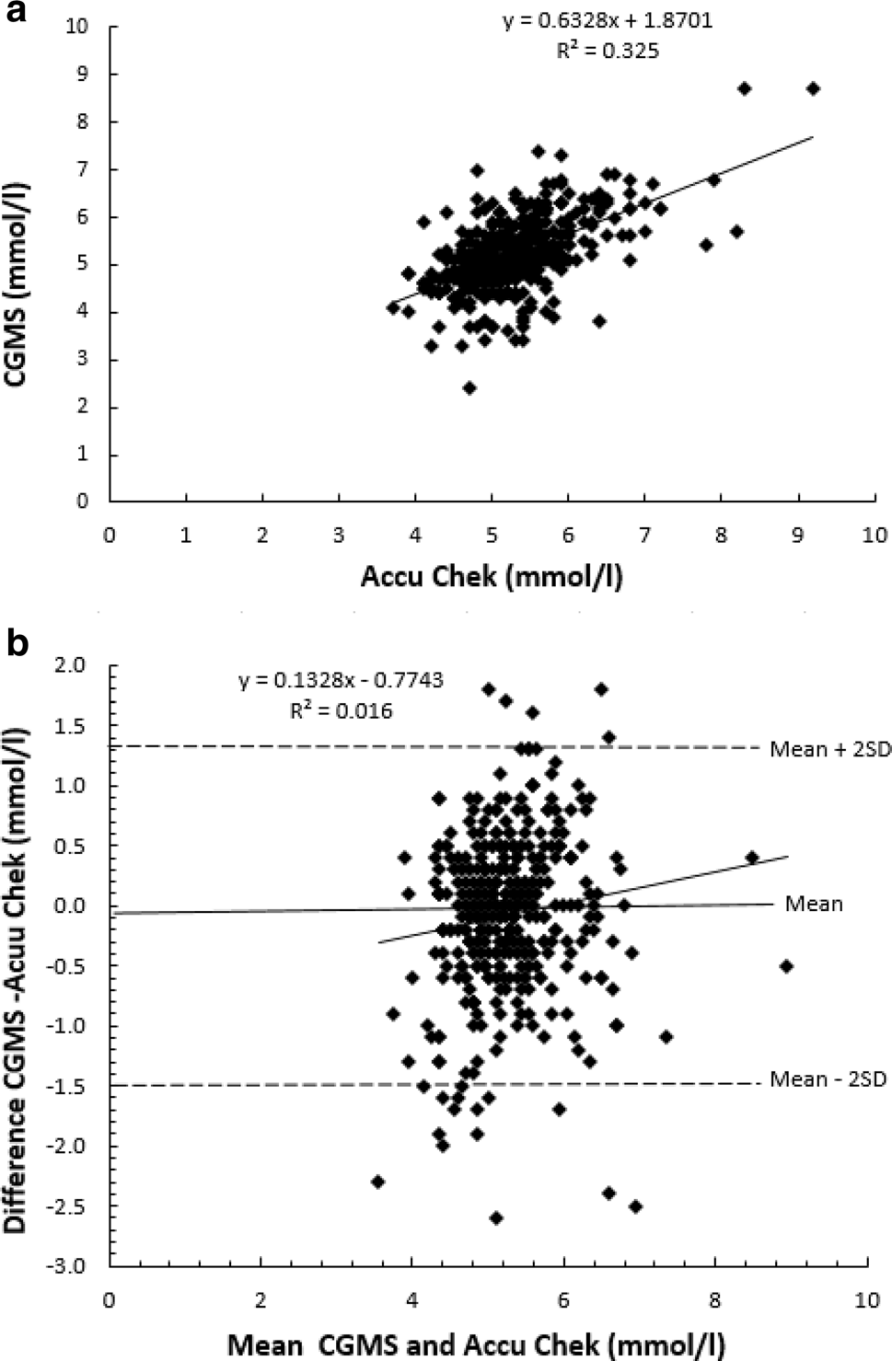
**a** Pearson regression of 405 paired glucose measurements between the Continuous Glucose Monitoring Systems (CGMS) and finger-prick blood glucose monitoring device (Accu-Chek) data. **b** Bland–Altman analysis of 405 paired glucose measurements between the CGMS and Accu-Chek data

The Bland–Altman analysis indicated a good agreement between the CGMS and Accu-Chek for glucose level for 405 paired readings (mean difference 0.1 mmol; 95% confidence interval − 0.166 to − 0.034; limits of agreement − 1.4 to 1.3 mmol). These results suggest that the data from the CGMS is reliable (Fig. 1).

### Blood glucose profile

**Fig. 2:**
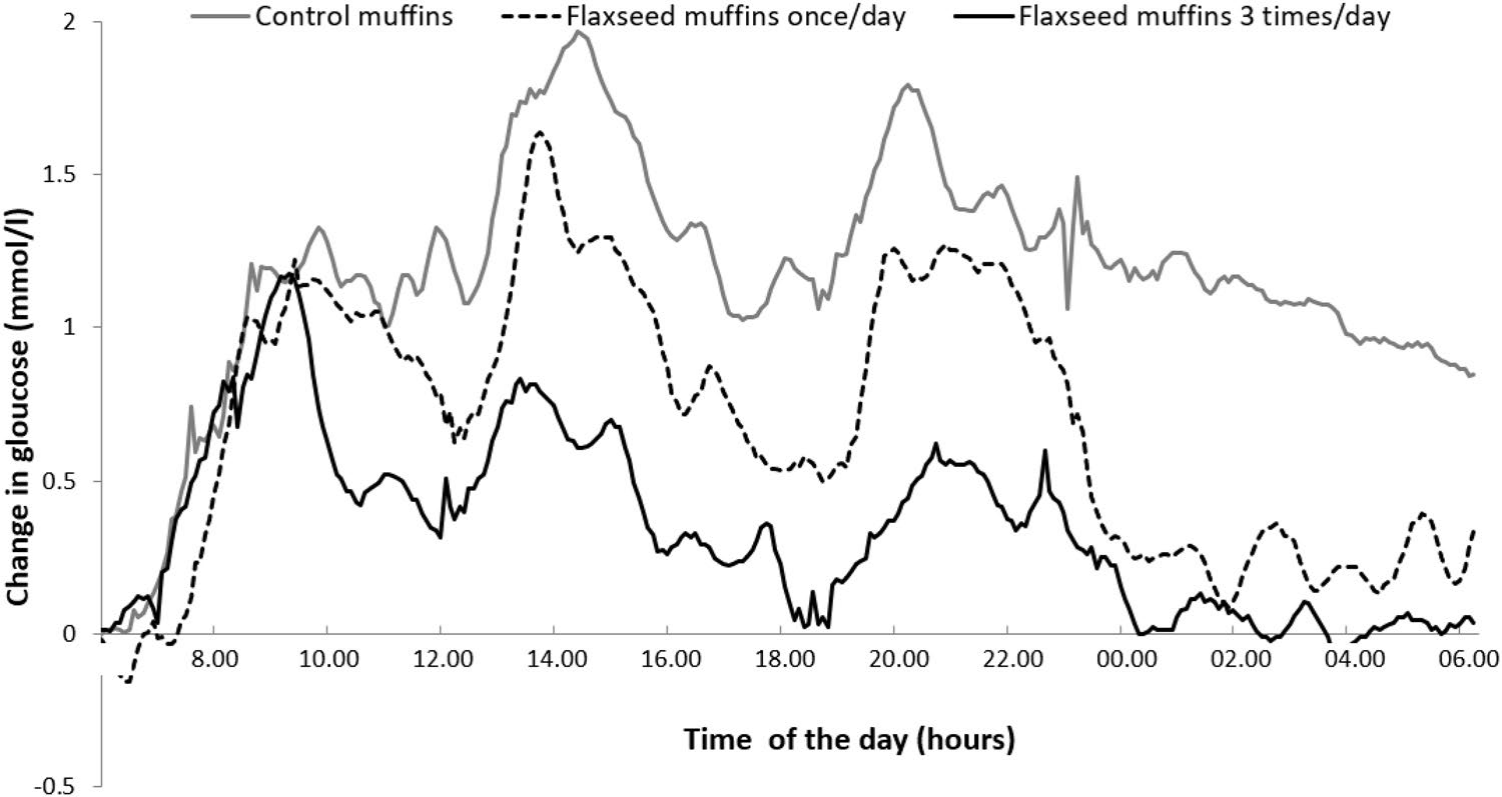
Mean change in blood glucose (mmol/l) during 24 h, for each test day (*n* = 15)

The blood glucose profile over 24 h for control muffins, once-a-day flaxseed muffins and three-times-a-day flaxseed muffins is presented in (Fig. 2). All the investigations showed the same trend: splitting the portion of flaxseed to three times a day produced the lowest results, followed by flaxseed muffins once a day, while the control muffins had the highest glucose levels.

### Blood glucose iAUC

The blood glucose iAUC results (Table 3) show that adding flaxseed muffins to the diet whether in one portion or split over three times a day, had the effect of lowering the blood glucose iAUC over 24 h, this was significant between the control muffins and flaxseed three times a day (*p* = 0.014). During the night there were significant differences between the control muffins and both flaxseed treatments (flaxseed once a day and flaxseed three times a day) (*p* = 0.005). There was no significant difference between the flaxseed treatments.

**Table 3:** Blood glucose iAUC for all (24 h) and sections (daytime, morning, afternoon and evening, night-time) of each test day (mean ± SD)

	Control muffins (mmol/l)	Flaxseed muffins once/day (mmol/l)	Flaxseed muffins 3 times/day (mmol/l)	*P* values
24 h iAUC (6.00–6.00 h)	1760.1 ± 1087.7	1214 ± 1065.4	893.8 ± 692.8^a^	0.014
Daytime iAUC (6.00–22.00 h)	1207.0 ± 728.2	931.7 ± 787.6	689.6 ± 448.4	0.127
Morning iAUC Day (6.00–12.00 h)	312.8 ± 183.9	287.4 ± 245.0	224.4 ± 139.8	0.627
Afternoon-evening iAUC Day (12.00–22.00 h)	768.8 ± 478.3	648.9 ± 564.2	470.3 ± 352.8	0.057
Night-time iAUC (22.00–6.00 h)	553.1 ± 382.3	282.3 ± 292.4^a^	204.2 ± 276.5^a^	0.005

^a^Significant difference (*p* < 0.05) compared to control muffins

### Absolute blood glucose level

The results for the absolute blood glucose level for each test day (control muffins, flaxseed muffin once per day and flaxseed muffins three times per day) over 24 h, over the daytime and over the night-time are shown in (Table 4). There were no significant differences in absolute blood glucose between the test days for any examined time period, except for the night (22.00–6.00 h) where there was a significant reduction (*p* = 0.011) in blood glucose concentration after consuming flaxseed once (4.8 ± 0.3 mmol/l) compared to the control muffins (5.2 ± 0.1 mmol/l).

**Table 4:** Average blood glucose concentration (mmol/l) for all (24 h) and sections (daytime, morning, afternoon and evening, night-time) of each test day (mean ± SEM)

	Control muffins (mmol/l)	Flaxseed muffins once/day (mmol/l)	Flaxseed muffins 3 times/day (mmol/l)	*P* values
24 h (6.00–6.00 h)	5.2 ± 0.1	5.1 ± 0.1	5.2 ± 0.1	0.419
Day time (6.00–22.00 h)	5.2 ± 0.1	5.3 ± 0.1	5.4 ± 0.1	0.436
Morning (6.00–12.00 h)	4.9 ± 0.2	5.0 ± 0.2	5.3 ± 0.1	0.175
Afternoon-evening (12.00–22.00 h)	5.5 ± 0.1	5.4 ± 0.1	5.4 ± 0.1	0.745
Night-time (22.00–6.00 h)	5.2 ± 0.1	4.8 ± 0.3^a^	5.0 ± 0.1	0.011

^a^Significant difference (*p* < 0.05) compared to control muffins

### Blood glucose peak values and time to peak

The peak and time to peak of each of the three meals consumed during the day (Table 5) were not significantly different between the test days.

**Table 5:** Blood glucose peak and time to peak for each main meal for each test day (mean ± SD)

	Control muffins	Flaxseed muffins once/day	Flaxseed muffins 3 times/day	*P* values
Peak breakfast (mmol/l)	6.4 ± 0.8	6.0 ± 0.8	6.4 ± 1.0	0.207
Peak lunch (mmol/l)	6.4 ± 0.8	6.4 ± 1.0	6.4 ± 0.8	0.920
Peak dinner (mmol/l)	6.3 ± 0.4	6.4 ± 0.9	6.1 ± 0.7	0.307
Time to peak breakfast (min)	9.52 ± 1.5	9.50 ± 1.3	9.57 ± 1.6	0.970
Time to peak lunch (min)	15.00 ± 2.0	14.37 ± 1.7	14.45 ± 0.1	0.420
Time to peak dinner (min)	18.27 ± 6.6	19.50 ± 4.1	20.00 ± 4.7	0.169

### Gastrointestinal symptoms

**Fig. 3:**
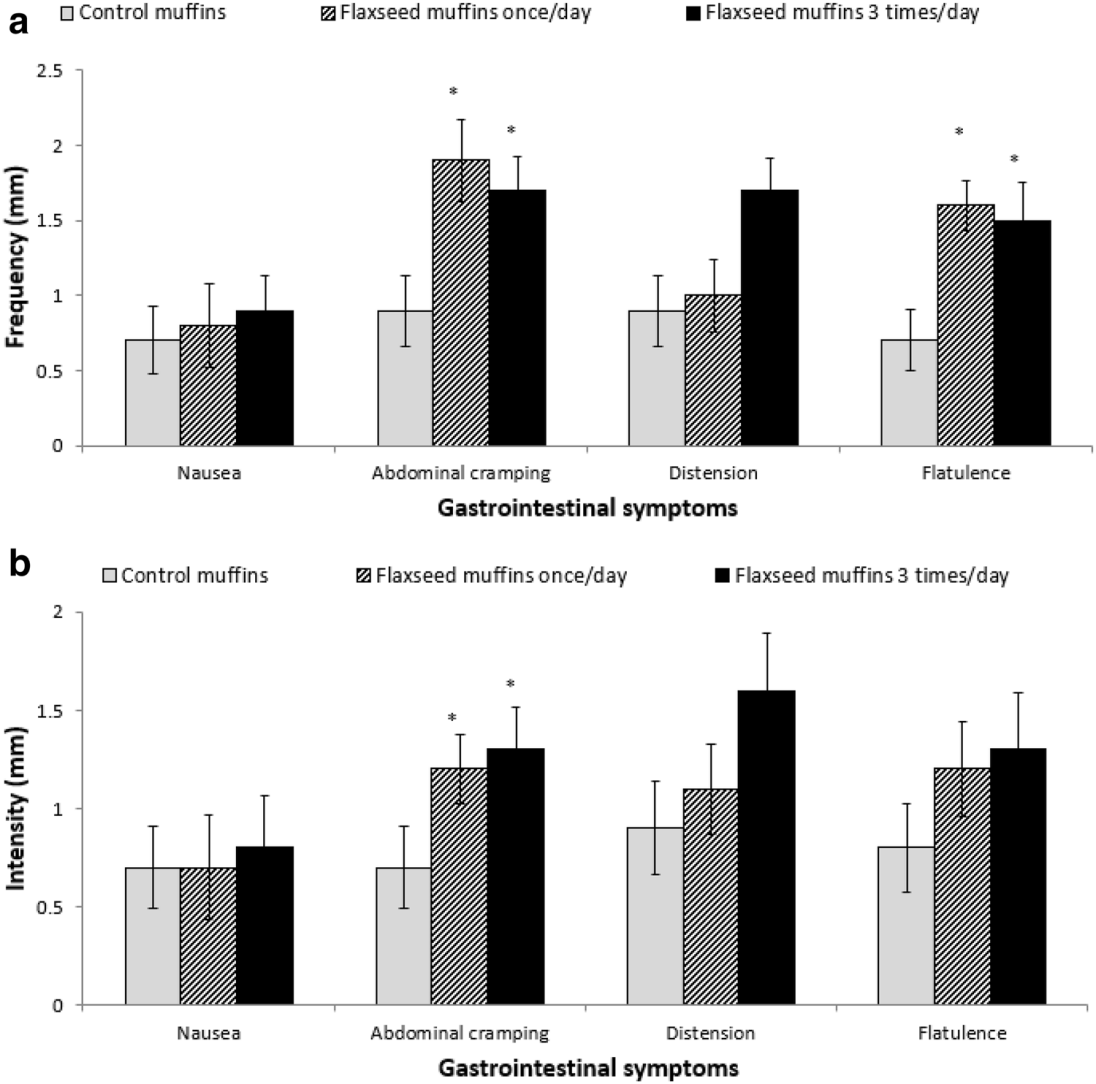
Mean frequency (**a**) and intensity (**b**) of some gastrointestinal symptoms 24 h post consumption for each test day (*n* = 15). *Significant difference (*p*<0.05) compared to control muffins

The effect of adding flaxseed to muffins on gastrointestinal symptoms 24 h post consumption was investigated and the results (Fig. 3) for the frequency and intensity for all the symptoms were similar for both flaxseed treatments. There was significant differences between both flaxseed treatments and the control muffins (*p* < 0.001) for the frequency and intensity of abdominal cramping and frequency of flatulence.

## Discussion

The present study aimed to investigate the effect of adding flaxseed to muffins in one single portion, or split into three portions consumed throughout the day, on blood glucose profile using CGMS among healthy participants. The results of this study demonstrated that flaxseed muffins given 3 times a day were effective at lowering and maintaining blood glucose levels over 24 h, compared to the control muffins and that both flaxseed treatments resulting in a lower blood glucose iAUC during the night. To the best of our knowledge there is currently no previous study that has investigated the effect of flaxseed on blood glucose response over an entire 24-h period.

Reliable evidence has shown that the consumption of low GI food reduces large blood glucose fluctuations [2, 5]. It has been deduced from various studies that low GI foods ingested at breakfast reduce glycaemia after a subsequent standardised lunch; a phenomenon known as the “second-meal effect”. In the short and medium terms, the consumption of food which causes a second-meal effect may assist in sustaining low blood glucose concentrations, consequently reducing demands upon the insulin-mediated blood glucose regulatory systems [5]. This implies that first meal of the day (breakfast) may be the largest contributor to daily glycaemic control, which can result in improvements in glucose tolerance for subsequent meals such as lunch and dinner [32, 33] if low GI foods are consumed. A possible mechanism is that breakfast may serve as primer to the insulin-sensitive tissues either by: (1) adjusting β-cell responses with changed incretin signalling or (2) an insulin sensitivity change following breakfast [34] as several studies have demonstrated greater insulin sensitivity in the morning than there is in the afternoon [35]. Although the results of the current study did not show differences after each of the individual meals, this effect is applicable to the current study as the results of iAUC over 24 h was significantly lower when flaxseed muffins were consumed once a day, as well as when they were split across the day, compared to the control.

Many studies have looked at the effect of low GI foods on the blood glucose profile over 24 h among healthy participants [2, 19, 21, 22]. The blood glucose profile over 24-h for the above studies showed lower values following low GI food, especially during the night which is consistent with the results of the current study. The results for blood glucose iAUC during the night indicated that both flaxseed treatments produced lower readings than the control muffins. This is an important finding as much of the research to date on flaxseed has focused on the postprandial period; however, this demonstrates an improved hepatic insulin sensitivity, effectively decreasing hepatic glucose output throughout the night from just the consumption of either one single 30 g dose of flaxseed or three 10 g doses dispersed throughout the day.

Studies have shown that low GI diets in healthy adults can significantly lower fasting plasma glucose levels, which suggests that by switching to a low GI diet an improvement in hepatic insulin sensitivity that would effectively decrease the hepatic glucose output [19]. If these changes are applied over the long term they could potentially improve glucose control, which could result in a reduced chronic disease risk such T2D and CVD [21]. The impact of the reduction of blood glucose is a basic element of a low GI diet and the mechanisms underlying the metabolic impacts are multifactorial including glucose absorption, a reduced gastric emptying rate, the food matrix, a slower small-intestinal transit as well as a decreased insulin response [36].

Flaxseeds contain many components known to impact blood glucose. It is well established that soluble dietary fibre can impact blood glucose control through delayed absorption within the gastrointestinal tract [37, 38]. The flaxseed muffins contained 4.9 g/100 g of fibre, which could be one of the main reasons for the changes in blood glucose seen in the current study. However, a recent study compared the intake of flaxseed or psyllium for 12 weeks and found flaxseed to have superior impacts on glycaemic response [15] suggesting that other components may also contribute to the reduction in blood glucose. Flaxseed contains n-3 fatty acids alpha-linolenic acid has which has previously been shown to have a positive impact upon glycaemic control [37, 38]. A previous systematic review and meta-analysis [14] showed that there was a significant association between flaxseed supplementation and a reduction in blood glucose; however, when subgroup analysis was completed the significant reduction in blood glucose were found only in studies using whole flaxseed but not flaxseed oil and lignan extract. However, the flaxseed oil was approaching significance (*p* = 0.08). Although the systematic review by Mohammadi-Sartang et al. [14] highlighted that flaxseed lignan extract had no significant impact on blood glucose, plant lignan has been suggested to be able to lower the risk of cardiovascular diseases as well as inhibit the effect of developing type two diabetes by reducing inflammatory response and insulin resistance [39]. It has been shown that flaxseed lignan secoisolariciresinol diglucoside restricts expression of the phosphoenolpyruvate carboxykinase gene, which is considered the main enzyme in glucose synthesis in the liver [40, 41]. The richest source of plant lignans is flaxseed which incorporates approximately 75–800 times more lignans in comparison to cereal grains, legumes, fruits and vegetables [9]. Finally, the protein in the flaxseed may also cause an increased insulin secretion resulting in decreased blood glucose [42]. It is likely that the reductions in blood glucose observed in the current study are due to the cumulative effects of all of these components and their associated mechanism; however, further clinical trials are required to confirm this.

The amount of flaxseed added to the muffins in the current study was based on a study by Wu et al. [43], who found that 30 g of milled flaxseed was effective in lowering blood glucose. Lipilina and Ganji [44] also demonstrated that replacing 50% of the flour with ground flaxseed resulted in the most acceptable sensory evaluation compared to the control bread with wheat flour. In this study 30 g of flaxseed equalled a 48% replacement of flour. Ground flaxseed has also been shown to enhance the sensory attributes of muffins and could be added in high amounts (up to 50%) without any negative effects [45]. Due to a recent report from the European Food Safety Authority (EFSA), there have been some concerns about flaxseed due to the presences of cyanogenic glycosides, with many media outlets reporting that their intake should be limited especially in infants [46]. EFSA have now clarified this misconception. They highlight that 14.7 g of flaxseed per day could reach the Acute Reference Dose. The Acute Reference Dose refers to an estimate of a daily oral exposure for an acute duration (24 h or less) to the human population that is likely to be without an appreciable risk of deleterious effects during a lifetime [47]. EFSA also highlight that there is overall uncertainty with the present assessment, and it is more likely to overestimate than to underestimate the risk. However, future studies should examine smaller doses and the implications this can have on 24-h glycaemic responses, particularly as flaxseeds are most often consumed with or as apart of breakfast. Furthermore, the extent of granularity of the flaxseed was not measured in the current study and little is known about how the degree of granularity effects glycaemic response and bioavailability of flaxseed nutrients.

The current study also investigated gastrointestinal symptoms compared with the control muffins and found that flaxseed muffins of both treatment regimens caused some side effects on the gastrointestinal system. This is consistent with the results of Kristensen et al. [48], who found that 5 g flaxseed dietary fibre supplementation for 12 weeks (with or without the addition of calcium) resulted in some gastrointestinal symptoms, especially abdominal pain, flatus and diarrhoea, which may be due to the amount of dietary fibre in the flaxseed. These results suggest flaxseed muffins can cause some gastrointestinal side effects, although they were not severe as the highest mean value was only 1.9 on a scale of 1–10.

## Conclusions

The results of this study indicated that adding flaxseed to the diet can produce a lower glucose profile over 24 h in a free-living context compared to the control muffins. Furthermore, flaxseeds were effective in improving the blood glucose profile over the 24 h; however, to improve blood glucose both over 24 h and overnight flaxseeds are better consumed in smaller amounts dispersed throughout the day. This study provides important data about adding flaxseed to the diet for healthy individuals, which can improve glycaemic response throughout the day and overnight.
